# Preventing depression in aphasia: A cluster randomized control trial of the Aphasia Action Success Knowledge (ASK) program

**DOI:** 10.1177/17474930231176718

**Published:** 2023-06-05

**Authors:** Brooke Ryan, Ian Kneebone, Miranda L. Rose, Leanne Togher, Emma Power, Tammy Hoffmann, Asaduzzaman Khan, Nina Simmons-Mackie, Marcella Carragher, Linda Worrall

**Affiliations:** 1 Discipline of Clinical Psychology, University of Technology Sydney, Ultimo, NSW, Australia; 2 Centre of Research Excellence in Aphasia Rehabilitation Recovery, La Trobe University, Bundoora, Victoria, Australia; 3 Queensland Aphasia Research Centre, The University of Queensland, Brisbane, Queensland, Australia; 4 Curtin School of Allied Health, Curtin University, Perth, WA, Australia; 5 School of Allied Health, Human Services and Sport, La Trobe University, Bundoora, VIC, Australia; 6 Department of Communication Sciences, Sydney School of Health Sciences, Faculty of Medicine and Health, The University of Sydney, Sydney, NSW, Australia; 7 Speech Pathology, Graduate School of Health, University of Technology Sydney, Ultimo, NSW, Australia; 8 Institute for Evidence-Based Healthcare, Faculty of Health Sciences & Medicine, Bond University, Gold Coast, QLD, Australia; 9 Southeastern Louisiana University, Hammond, LA, USA

**Keywords:** Aphasia, stroke, depression, psychosocial intervention, prevention, cluster randomized controlled trial

## Abstract

**Background:**

Stroke patients with aphasia and their caregivers have higher incidence of depression than those without aphasia.

**Aims:**

The objective of the study is to determine whether a tailored intervention program (Action Success Knowledge; ASK) led to better mood and quality of life (QoL) outcomes than an attention control with a 12-month end point at cluster and individual participant level.

**Methods:**

A multi-site, pragmatic, two-level single-blind cluster randomized controlled trial compared ASK to an attention control (secondary stroke prevention program). Ten metropolitan and 10 non-metropolitan health regions were randomized. People with aphasia and their family members were recruited within 6 months post-stroke who scored ⩽12 on the Stroke Aphasic Depression Questionnaire Hospital Version–10 at screening. Each arm received manualized intervention over 6–8 weeks followed by monthly telephone calls. Blinded assessments of QoL and depression were taken at 12 months post-onset.

**Results:**

Twenty clusters (health regions) were randomized. Trained speech pathologists screened 1744 people with aphasia and 373 participants consented to intervention (n = 231 people with aphasia and 142 family members). The attrition rate after consent was 26% with 86 and 85 participants with aphasia in the ASK arm and attention control arm, respectively, receiving intervention. Of those 171 who did receive treatment, only 41 met the prescribed minimum dose. Multilevel mixed effects modeling under the intention-to-treat protocol showed a significant difference on the Stroke and Aphasia Depression Questionnaire–21 (SADQ-21, N = 122, 17 clusters) in favor of the attention control (β = –2.74, 95% confidence interval (CI) = –4.76 to –0.73, p = 0.008). Individual data analysis using a minimal detectable change score for the SADQ-21 showed the difference was not meaningful.

**Conclusion:**

ASK showed no benefit over attention control in improving mood and preventing depression in people with aphasia or their family members.

Stroke patients with aphasia have a higher incidence of depression (62–70%) than stroke survivors without aphasia, which is similar to their caregivers who have increased risk of depression over time.^1–3^ Previous research has found that depressive symptoms, language functioning and functional communication predict quality of life (QoL) in aphasia.^4^ Our longitudinal investigation (n = 58 participants with aphasia) of the first year post stroke showed low mood had a negative effect on life with aphasia^5^ and was a potential modifiable factor that would improve outcomes. Since depression is prevalent and negatively impacts QoL after aphasia, prevention and treatment of depression are a high priority.

Selective serotonin reuptake inhibitors (SSRIs) have been shown to reduce the risk of future depression post stroke but also lead to an increase in the risk of seizures and bone fractures and are therefore not recommended for routine post-stroke use.^6^ Our systematic review found no effective non-pharmacological interventions that prevent depression in aphasia, but found that mood can be improved by multimodal individual goal setting and family support.^7^ Hence, this study evaluated an intervention designed to prevent depression in people with aphasia and their family members. The specific aim was to determine whether a tailored, early intervention program, Aphasia Action Success Knowledge (ASK), led to better mood and QoL outcomes in people with aphasia, than an attention control (Secondary, Stroke Prevention Information Program, SSPIP) 12 months post-stroke at the cluster and individual level for patients with aphasia and their family members:

*Hypothesis 1.* Patients with aphasia who receive the ASK program will have significantly better outcomes on the *Stroke and Aphasia Depression Questionnaire–21 (SADQ-21)*^8^ and the *Assessment for Living with Aphasia (ALA)*^9^ at 12 months post stroke than those who receive an attention control program.
*Hypothesis 2*. Caregivers of patients with aphasia who receive the ASK program will have significantly better outcomes on the *Bakas Caregiving Outcomes Scale (BCOS)*^10^ and the *General Health Questionnaire–28 (GHQ-28)*^11^ at 12 months post stroke than those who receive an attention control program.

Health regions were chosen as clusters to reduce the risk of treatment contamination and to enable provision of study interventions alongside usual care for the study duration (i.e. across multiple health facilities within the first year post-stroke).

## Methods

### Design

Ethics approval was obtained from the University of Queensland and all study sites (HREC/14/QTDD/30). The multicentre cluster randomized controlled trial (cRCT) protocol was published.^12^ Design and reporting was guided by CONSORT reporting guidelines for non-pharmacological cRCTs,^13^ and TIDieR checklist.^14^ Since protocol publication, eligibility criteria were revised to assist participant recruitment. Exclusion criteria of pre-stroke aphasia or previous depression were removed and the cut-off score on the depression screening tool (Stroke Aphasic Depression Questionnaire–10 (SADQH-10))^15^ was increased from 9 to 12. An embedded process evaluation including a study of treatment fidelity^16^ will be reported later.

### Participants: Recruitment and selection criteria

Clusters were 20 self-selected health regions across Australia recruited through the Speech Pathology Email Chat Google group. Cluster eligibility criteria included provision of aphasia rehabilitation services across the period of intervention; potential clusters were excluded if they were participating in other clinical trials that might conflict with recruitment or intervention. Equal number of clusters from metropolitan and non-metropolitan regions were sampled to cater for any geographical psychology service provision differences, with the aim to recruit 20 people per cluster with post-stroke aphasia. Clusters were randomly allocated in progressive blocks (2 capital, 2 noncapital) by the blinded statistician (A.K.) to either the experimental arm (the ASK program) or the similarly formatted and delivered attention control arm (SSPIP), using a computer-generated random number scheme. Sequential numbers were assigned to each cluster to ensure allocation concealment. Allocation concealment from cluster site staff was not possible as each of the intervention arms had different content. Participants with aphasia, their family, and outcome assessors were blinded to intervention allocation.

Sample size calculations were calculated for both primary outcome measures (ALA^9^ and SADQ-21^8^). Power calculations on the ALA^9^ were calculated from an intensive aphasia treatment study^17^ and an Australian longitudinal aphasia study.^5^ Power calculations on the SADQ21^8^ were calculated from the Communication and Low Mood (CALM) study.^18^ The ALA required a larger sample size compared to the SADQ-21,^8^ and therefore, the larger sample size required by the ALA^9^ was determined necessary to adequately power the study. To achieve a power of 80% with a 5% level of significance in comparing the two arms of the study (Aphasia ASK vs attention control—SSPIP), we needed 186 patients (93 per arm) with a standardized effect size of 0.367, computed using ALA data.^5^ The extent to which power was diminished by clustering was considered in relation to the design effect (DE) = 1 + (m − 1) r10, where m = the average size of a cluster and r is the intra-class correlation coefficient. Typically, intraclass correlation coefficients are small (<0.02); thus, a conservatively estimated intra-class correlation of 0.02 was used. A cluster size of 20 was chosen based on the feasibility of running the intervention and the availability of patients with aphasia within clusters. Thus, DE = 1 + (20 − 1) × 0.02 = 1.38, and the total sample size required was calculated as 186 × 1.38 ≈ 258. To account for an attrition rate of 25% to the 12-month follow-up period, 344 patients would be needed (172 per arm).

All people with aphasia after stroke admitted to the participating health region were eligible to be screened for inclusion. They were diagnosed by a qualified speech pathologist using the Western Aphasia Battery−Revised^19^ and clinical judgment. They were screened using a case history and one of the study designated mood screens, conducted either in the hospital or at an outpatient clinic in the first 6 months post-stroke. Exclusion criteria included concomitant cognitive disorders (e.g. dementia, primary progressive aphasia), aphasia with an etiology other than stroke, current psychiatric diagnosis (e.g. depressive disorder; confirmed by medical record), current depressive symptoms upon screening with SADQ-10 (score of ⩾12)^8^ or the Depression Intensity Scale Circles (score of ⩾3),^20^ currently receiving treatment in a psychiatric setting, or enrolment in other aphasia or depression clinical treatment studies. Adult family members were invited to participate although those with a cognitive disorder were excluded. Pre- and post-data collection were undertaken by a blinded, independent speech pathologist at a location convenient to participants. Intervention was at a location convenient to participants, usually hospital or home.

### Procedure

A total of 252 speech pathologists were trained with 44 trained in the screening process, 177 trained in the delivery of their allocated intervention, and 31 trained in the outcome assessment. Speech pathologists were trained to deliver one intervention and in screening, consent, minimal dose, and reporting adverse events. Training was 5–6 h either in person or online by trial managers using a PowerPoint slide deck. Refresher training was available any time.

Potential participants were given an aphasia-friendly written participant information sheet and a verbal explanation of the research. Where required, capacity to consent was judged by trial speech pathologists using supported communication principles. Once consented, participants nominated their family members to be involved. If the family member agreed, the speech pathologist obtained their informed consent. See supplementary materials for the consent process. Participants were assessed by a blinded assessor by 6 months post stroke onset. Intervention was initiated within 2 weeks of baseline assessment. Face-to-face intervention was once a week for 1 h in person until selected module completion. Afterward, the treating speech pathologist contacted participants via monthly phone calls. Three modules (3 h) plus four phone calls (2 h) were the prescribed minimum intervention. At 12 months post-stroke, participants were re-assessed by a blinded assessor.

### Outcome measures

The two primary measures were the *SADQ-21*,^8^ a proxy-reported measure of observed depressive symptoms, and the *ALA*,^9^ an accessible patient-reported outcome measure of QoL. Secondary measures were self-reported stroke risk-related behaviors via a bespoke 10-item measure and for the family members, the *Bakas Caregiving Outcomes Scale–Revised*^10^ and the *GHQ-28*.^11^ Outcomes were assessed 2 weeks after randomization (pre-intervention) and 12 months post-stroke post intervention; hence, the primary endpoint was at 12 months post stroke.

### Interventions

Both programs include eight aphasia-friendly written booklets that the therapist, person with aphasia, and their family member used to guide discussion. Two booklets focused on setting goals while one booklet summarized the program and discussed goal progress.

The ASK program was developed from research regarding living successfully with aphasia.^21,22^ Intervention development used the three-phase process for developing and evaluating complex interventions described by Kirkevold et al.^23^ and based on UK Medical Research Council recommendations. A phase I/II study^24^ showed that ASK results were promising.

The attention control program was devised using the criteria of equivalence, distinctiveness, and attractiveness.^25^ The control intervention was equivalent for dose, format, and delivery but distinct in that the information was about secondary stroke prevention rather than maintaining psychological health. For more information about the interventions, see Supplementary Table 1 Intervention characteristics.

### Analysis

The Statistical Analysis Plan was finalized on 29 April 2021 prior to analysis, uploaded to the ANZCT site and is available by request. A multilevel mixed-effects model, which took into account participants being nested within clusters, examined whether changes in the continuous outcomes of interest varied over time and across the two groups, after adjustment for potential confounders. The model included fixed effects for the treatment group, time-point, and the treatment-by-time-point interaction. For the one binary outcome of whether participants were depressed on not using the SADQ-21,^8^ mixed effects logistic regression was used to examine the effects of the intervention. Covariates were at an individual level, reflecting differences between the two treatment arms and studies that showed an influence on our primary outcomes. They included age, gender, educational level, change in living situation during the intervention, discontinuation of usual care speech pathology services during the intervention period, walking status at the start of the intervention, and baseline severity of aphasia. Collinearity of the covariates was assessed before including them in the multivariable modeling. Attrition patterns across the two groups were examined to determine randomness of missing data and, if required, multiple imputation was implemented. In the assessment of the hypotheses, missing data were assumed as missing at random (MAR), which was assessed with a missing data sensitivity analysis. The residuals of the fitted models were examined to ensure that all required assumptions were met. An alpha level of 0.05 was accepted as significant. No adjustment for multiple comparisons was made to reduce the risk of type I error. Instead, we present the actual p values and 95% confidence intervals so that readers can interpret the results.

A per-protocol analysis included only those participants who had completed three or more face-to-face sessions, had both pre-and post-measures collected, and had intervention delivered by a therapist who passed the treatment fidelity check. Adherence to the phone calls was not considered in the per-protocol analysis as there was little compliance. To determine whether significant differences were meaningful, individual participant data analysis was undertaken^26^ using the most relevant psychometric data from Sutcliffe and Lincoln.^8^ For the SADQ-21, a minimal detectable change (MDC) value of 9.45 was used.

## Results

**Figure 1.**
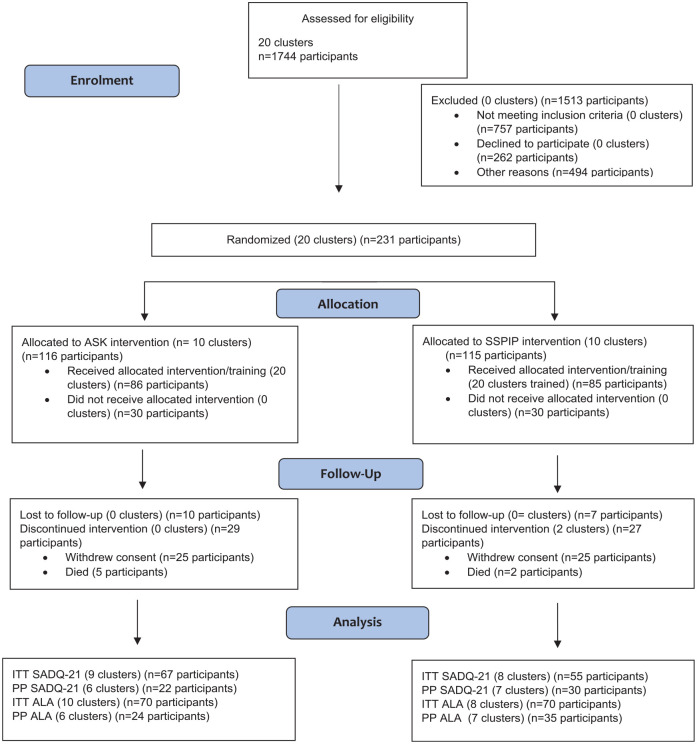
CONSORT flow diagram.

Recruitment occurred between 2015 and 2019. Figure 1 shows the CONSORT flow chart. Exclusion reasons are provided in Supplementary Table 3. A total of 231 people with aphasia, and 142 of their family members, from the 20 clusters consented. Target recruitment was not met with recruitment ending when funds were expended. There was a high attrition rate after consent with only 86 participants with aphasia in the ASK arm receiving intervention and 85 in the attention control arm. Of those 171 who did receive treatment, 41 met the prescribed minimum dose previously outlined^7^ of three modules completed and total contact time of 3 h plus 4 phone calls completed and total time of 2 h.

The number of participants and clusters in the intention-to-treat (ITT) analysis reduced to n = 122, 17 clusters for the SADQ-21^8^ and n = 140, 18 clusters for the ALA.^9^ Even fewer were included in the per-protocol analysis with n = 52, 13 clusters for the SADQ-21^8^ and n = 59 participants, 13 clusters for the ALA.^9^

Individual participant characteristics for both intervention groups are shown in Table 1.

**Table 1.** Participant characteristics.

	Control group (SSPIP) n (%)	Experimental group (ASK) n (%)	All
Clusters/health districts			
Capital City	5 (25)	5 (25)	10 (50)
Participants with aphasia, n	88	93	181
WAB-R-AQ (mean, SD)	77.52 (19.68)	75.52 (21.4)	76.49 (20.55)
Mild > 76	57 (64.77)	52 (55.91)	109 (60.22)
Moderate 51–75	19 (21.59)	29 (31.18)	48 (26.51)
Severe 26–50	9 (10.22)	6 (6.45)	15 (8.29)
Very severe < 25	1 (1.13)	4 (4.30)	5 (2.76)
Female	39 (44.32)	45 (48.38)	84 (46.41)
Age, mean (SD) range	66.84 (12.32) 37–92	67.32 (12.77) 30–93	67.09 (12.52) 30–93
Education 12 years or more	54 (61.36)	54 (58.06)	108 (59.69)
Language other than English	4 (4.55)	3 (3.22)	7 (3.87)
Employed	12 (13.63)	9 (9.68)	21 (11.60)
Living in community	39 (44.31)	39 (41.93)	78 (43.09)
Marital status			
Married/de facto	54 (61.36)	53 (56.99)	107 (59.11)
Widowed or separated	28 (31.81)	53 (56.99)	62 (34.25)
Never married	4 (4.54)	5 (5.38)	9 (4.97)
Walking independently	76 (86.36)	81 (87.09)	157 (86.74)
Days post stroke, mean (SD) range	115 (53.6) 16–210	96.93 (52.2) 17–200	103.80 (53.20) 16–210
Prescribed antidepressants	11 (14.86)	5 (5.26)	16 (9.47)
Family members	47	87	134
Relationship			
Spouse	34 (72.34)	50 (57.47)	84 (62.68)
Adult child	10 (21.27)	28 (32.18)	38 (28.35)
Other	3 (6.38)	9 (10.35)	12 (8.95)
Female	32 (68.08)	58 (66.66)	90 (66.16)
Age, mean (SD) range	59.18 (15.52) 20–87	56.69 (15.52) 19–83	57.50 (15.50) 19–87

Using data collected at baseline for any consenting participant (attrition occurred between consent, baseline, and intervention).

SSPIP: Secondary, Stroke Prevention Information Program; ASK: Action Success Knowledge; WAB-R-AQ: Western Aphasia Battery–Revised Aphasia Quotient; SD: standard deviation.

Usual care interventions are described in Supplementary Table 2.

Table 2 shows primary and secondary outcome measures pre- and post-intervention group comparison. Both interventions showed on average a decrease in depressive symptoms on the primary outcome measure, the SADQ-21,^8^ but also showed that the ASK group had more people who were depressed in the intervention period. While there were 1.1% fewer participants with aphasia scoring in the depressed range after the attention control program, there were slightly more (0.6%) scoring in the depressed range after the ASK intervention.

**Table 2.** Primary and secondary outcome measures pre- and post-intervention group comparison.

	Control group (SSPIP) Pre-intervention	Control group (SSPIP) Post-intervention	Experimental group (ASK) Pre-intervention	Experimental group (ASK) Post-intervention
Primary outcome measures				
SADQ-21, n	76	60	90	72
SADQ-21, mean (SD) range	12.24 (8.31) 0–37	10.43 (8.03) 0–31	14.08 (7.79) 0–35	13.09 (8.13) 0–33
SADQ-21 < 18 (not depressed)	61 (78.9)	48 (80)	63 (70)	51 (69.4)
ALA, n	88	74	91	74
ALA, mean (SD) range	91.81 (22.22) 41–143	103.45 (21.12) 49–143	93.3 (21.41) 47–135	103.98 (21.69) 62–143
Secondary outcome measures				
SRB, n	74	74	74	74
SRB, mean (SD) range	7.80 (1.61) 3–10	8.2 (1.42) 4–10	7.8 (1.6) 4–10	8.16 (1.33) 5–10
GHQ-28, n	26	28	54	57
GHQ-28, mean (SD) range	23.96 (10.48) 8–50	22.21 (11.05) 7–52	22.55 (10.39) 3–49	22.64 (12.28) 5–66
BCOS, n	25	27	54	56
BCOS, mean (SD) range	55.84 (10.03) 27–66	60.0 (12.97) 21–83	56.77 (11.61) 29–84.27	59.01 (16.01) 24–102

Using imputed data for missing items.

ASK: Action Success Knowledge; SADQ-21: Stroke and Aphasia Depression Questionnaire–21 item (score range 0–63, higher scores = higher level of depression); SD: standard deviation; ALA: Assessment for living with Aphasia (score range 0–148, higher scores = better quality of life); SRB: self-reported stroke risk-related behaviors (score range 0–10, higher scores = more ideal behaviors); GHQ-28: General Health Questionnaire–28 (score range 0–36, higher scores = less desirable health outcomes); BCOS: Bakas Caregiving Outcomes Scale–Revised (score range 15–105, higher scores = more positive caregiver outcomes).

Multilevel mixed-effects modeling results for both analyses are reported on primary and secondary outcome measures (Table 3). After adjusting for a set of covariates, multilevel modeling under ITT protocol showed that average SADQ-21 score was 2.74 points lower for the attention control group compared to the experimental ASK group (β = –2.74, 95% confidence interval (CI) = –4.76 to –0.73, p = 0.008) at 12 months post-stroke. The attention control group had a 2.94-point lower average SADQ-21^8^ score than the experimental ASK group (β = –2.38, 95% CI = –4.71 to –0.07, p = 0.04) under the per-protocol analysis. The attention control program had fewer depressed symptoms reported on the SADQ-21^8^ compared to the ASK group. There were no significant differences in the ITT and per-protocol analyses between groups on the other primary outcome measure, the ALA.^9^

**Table 3.** Results of multilevel regression modeling^a^ examining the effect of the experimental (ASK) versus attention control (SSPIP) interventions on outcomes.

Outcomes	Intention-to-treat analysis β (95% CI), p value	Per-protocol analysis β (95% CI), p value
SADQ-21 Total *Attention Control (ref: ASK group)*	−2.74 (−4.76 to −0.73), 0.008** n = 122, 17 clusters	−2.38 (−4.71 to −0.07), 0.04* n = 52, 13 clusters
ALA Total *Attention Control (ref: ASK group)*	−0.39 (−5.0 to 4.19), 0.87 n = 140, 18 clusters	−4.2 (−.1.95 to 10.43), 0.18 n = 59, 13 clusters
SRRB Total *Attention Control (ref: ASK group)*	−0.09 (−0.41 to 0.23), 0.58 n = 141, 18 clusters	−0.18 (−0.27 to 0.64), 0.43 n = 58, 13 clusters
GHQ-28 Total *Attention Control (ref: ASK group)*	−3.19 (−6.45 to 0.08), 0.06 n = 82, 16 clusters	−1.01 (−5.90 to −3.87), 0.68 n = 33, 10 clusters
BCOS Total *Attention Control (ref: ASK group)*	2.60 (−1.66 to 6.84), 0.23 n = 76, 16 clusters	−0.19 (−7.23 to 6.85), 0.96 n = 31, 11 clusters

Per-protocol analysis for participants who had pre- and post-outcome measures, who completed 3 or more face-to-face sessions and where intervention was delivered by a therapist who passed the treatment fidelity check.

β: adjusted regression coefficient; CI: confidence interval; SADQ-21: Stroke and Aphasia Depression Questionnaire–21; ASK: Action Success Knowledge; ALA: Assessment for living with Aphasia; GHQ-28: General Health Questionnaire–28; BCOS: Bakas Caregiving Outcomes Scale–Revised; WAB-AQ: Western Aphasia Battery–Aphasia Quotient.

a Adjusted for age, gender, educational level, living situation, walking status, and WAB-AQ baseline measure at time 1.

* Significant at α < 0.05.

** Significant at α < 0.01.

There were no significant differences in the ITT analyses for the secondary outcome measures. Hence, neither hypothesis was supported in the between-group analyses.

A mixed-effects logistic regression using the cutoff score of 18 on the SADQ-21^8^ (depressed vs non-depressed) as the dependent variable (n = 170, 18 clusters) showed that participants in the attention control group had a 51% lower odds of developing depressive symptoms than their counterparts in the experimental ASK intervention group (OR = 0.49, 95% CI = 0.19 to 1.28, p = 0.15); however, the association proved non-significant.

For the SADQ-21^8^ significant result (n = 128), we calculated whether there were changes more than the MDC of 9.45:^8^ 101 participants (79%) showed no MDC, 15 (12%) showed a positive change, and 12 (9%) showed a negative change. Of the 12 people who became worse, 4 were in the attention control group and 8 in the ASK experimental group. Of the 15 people who improved, 7 were in the control group and 8 in the ASK experimental group.

There were no adverse events or serious adverse events attributable to the interventions (see Supplemental Table 4).

## Discussion

This cluster RCT showed no measurable benefit of ASK intervention, for improving mood and QoL outcomes for the person with aphasia and their family member. Both hypotheses were not supported. There was a significant difference between the interventions in favor of the attention control group on the primary outcome measure (SADQ-21,^8^ a report of depressive symptoms completed by family members). Individual participant data analysis^26^ showed the majority of participants did not change beyond the MDC score.

Several biases in the study methodology may have contributed to these results. The Catalogue of Biases by the Centre for Evidence-based Medicine (https://catalogofbias.org/biases/), major biases include an insufficient sample size bias (sample size of 181 instead of 344 required via power analysis), collider bias (the attention control intervention had a probable effect on outcome, the ASK intervention was more complex for participants to understand), and insensitive measure bias (both primary outcomes measures were self report). Another potential bias may include ascertainment bias (correct identification of individuals for recruitment to the study such that participants were included who did not have depression or were low mood). A usual care group would have indicated whether the addition of either intervention to usual care was significant.

The cluster RCT design may have limited the effectiveness of the trial, given the complexity of the intervention. Siebenhofer et al.^27^ found that 85% of cluster RCTs showed no results for complex interventions and Sutton et al.^28^ note that, while cluster RCTs are key to evaluating stroke interventions, there are barriers to conducting such studies. For example, in this trial a large number of participants were screened but ultimately under-recruitment occurred with potential for multiple sources of bias. Stepped wedge designs are recommended as an alternative.^28^

The ASK intervention alone may not have been sufficient to improve mood. ASK aimed for active discussion of psychosocial topics with planned activities between sessions that operationalized well-being strategies, supported by regular follow-up phone calls. Continued investigation of treatment fidelity and the process evaluation will shed light on important questions such as therapists’ experiences of recruitment and delivering the interventions. Future trials might consider approaches such as cognitive behavior therapy (CBT).^29,30^ Notably, evidence for preventing depression in other populations remains elusive.^31,32^ The importance of this topic mandates that further research is required to identify and evaluate effective interventions, and could include co-designing interventions, providing secondary stroke prevention information and discussion, and training speech pathologists in psychosocial education techniques and counseling.

## Supplemental Material

sj-docx-1-wso-10.1177_17474930231176718 – Supplemental material for Preventing depression in aphasia: A cluster randomized control trial of the Aphasia Action Success Knowledge (ASK) program Click here for additional data file. Supplemental material, sj-docx-1-wso-10.1177_17474930231176718 for Preventing depression in aphasia: A cluster randomized control trial of the Aphasia Action Success Knowledge (ASK) program by Brooke Ryan, Ian Kneebone, Miranda L. Rose, Leanne Togher, Emma Power, Tammy Hoffmann, Asaduzzaman Khan, Nina Simmons-Mackie, Marcella Carragher and Linda Worrall in International Journal of Stroke
