# Supplementary material for: Preventing depression in aphasia: A cluster randomized control trial of the Aphasia Action Success Knowledge (ASK) program
Source: Int J Stroke. 2023 Jun 5;18(8):996–1004. doi: 10.1177/17474930231176718 (PMC10507993; doi:10.1177/17474930231176718)
Supplement: sj-docx-1-wso-10.1177_17474930231176718 – Supplemental material for Preventing depression in aphasia: A cluster randomized control trial of the Aphasia Action Success Knowledge (ASK) program [file sj-docx-1-wso-10.1177_17474930231176718.docx]

Supplementary Table 1. Intervention characteristics

|  | **Received ASK intervention**  (n= 86) |  | **Received SSPIP intervention**  (n = 85) |
| --- | --- | --- | --- |
| **Face to Face modules** | | | |
| Your story n (%) | 50 (57%) | Stroke- what is it? n (%) | 66 (79%) |
| Living the learning n (%) | 71 (82%) | Stroke- What are the risk factors? n (%) | 75 (89%) |
| Finding the positive n (%) | 76 87%) | Understanding medications for stroke prevention n (%) | 63 (75%) |
| Not just words n (%) | 74 (85%) | Lifestyle changes to prevent stroke n (%) | 71 (85%) |
| Stay connected n (%) | 63 (72%) | Develop an action plan to prevent stroke n (%) | 41 (49%) |
| Total number of modules completed (mean, SD) | 5.6 (1.6) |  | 4.8 (2.6) |
| Total face to face minutes (mean, SD, range) | 351.9, 142.3, 12-170 |  | 256.9, 89.8, 15-105 |
| **Follow up phone calls** |  |  |  |
| Total number of phone calls (mean, SD) | 3.6, 2.5 |  | 4.6, 2.6 |
| Total phone call minutes (mean, SD) | 90.3, 105.8 |  | 89.0, 76.1 |
| Received intervention from therapist that passed fidelity criteria upon review (yes, %) | 51 (59%) |  | 53 (63%) |
| Met prescribed intervention dose (yes, %) | 17 (20%) |  | 24 (29%) |
| SSPIP, secondary stroke prevention program (attention control); ASK Action, Success, Knowledge (experiential intervention). Prescribed face to face minimum dosage was 3 modules completed and total contact time of 3 hours and phone calls was a minimum dosage of 4 phone calls completed and total time of 2 hours. The training program as well as the experimental ASK and attention control stroke prevention programs are available at https://www.latrobe.edu.au/research/centres/health/aphasia/resources | | | |

Supplementary Table 2 – Additional aphasia and psychosocial interventions (usual care)

|  | **SSPIP Control Arm** | **ASK Intervention Arm** | **All** |
| --- | --- | --- | --- |
|  | n= 82 | n= 81 | n=163 |
| Minutes of usual care aphasia management during the study intervention period  (Mean, SD, Range) | 841.06, 1254.11, 0-7775 | 731.50, 836.8, 0-4500 | 786.61, 1065.46, 0-7775 |
| Minutes of formal counselling by health professional during intervention (Mean, SD, Range) | 54.02, 171.9, 0-1160 | 62.7, 188.42, 0-1320 | 58.46, 179.78, 0-1320 |
| Minutes of peer support group attendance during intervention (Mean, SD, Range) | 220.41, 527.07, 0-3840 | 151.86, 393.34; 0 – 2880 | 186.34, 465.3; 0 -3840 |
| Participation in specific manualised stroke or aphasia programs e.g. My Stroke Journey, The STEPS Skills program (n=, % yes) | 15, 6.7% | 19, 23.5% | 34, 20.9% |
| SSPIP, secondary stroke prevention program (attention control); ASK Action, Success, Knowledge (experiential intervention)  NB.18 participants did not receive usual care | | | |

Supplementary Table 3 – Exclusion reasons

| *n=* | 1513 |
| --- | --- |
| declined consent | 201 |
| discharged from health service/catchment and not able to be provided intervention by trial therapists | 196 |
| unable to consent (with supported communication and adaption of written consent procedure) | 188 |
| required translator to participate | 165 |
| progressive neurological condition | 119 |
| current psych dx | 82 |
| current psych symptoms indicated with screening tool | 82 |
| for palliative care | 68 |
| aphasia as result of other reason (TBI, brain tumour) | 67 |
| aphasia resolved | 62 |
| declined screening/participation | 61 |
| not 6 months post-stroke | 42 |
| not first incidence of aphasia | 41 |
| deceased | 35 |
| inadequate hearing/vision | 32 |
| medical condition impacting mental health | 32 |
| history of recurrent depression | 32 |
| reason unknown | 7 |
| enrolled in other treatment study | 1 |

Supplementary Table 4. Adverse events and serious adverse events

|  | **SSPIP** | **ASK** | **All** |
| --- | --- | --- | --- |
| Adverse events |  |  |  |
| Serious adverse events |  |  |  |
| 0 | 75 (80.6%) | 64 (72.7%) | 139 (76.8%) |
| 1 | 13 (14%) | 12 (13.6%) | 25 (13.8%) |
| 2 | 2 (2.2%) | 9 (10.2%) | 11 (6.1%) |
| >2 | 3 (3.2%) | 3 (3.4%) | 6 (3.3%) |
| Deaths | 2 (1.1%) | 5 (2.8%) | 7 (3.9%) |
| SSPIP, secondary stroke prevention program (attention control); ASK Action, Success, Knowledge (experiential intervention) | | | |

All research and site staff were responsible for the detection and documentation of events meeting the criteria and definition of an adverse event (AE) or a serious adverse event (SAE) according to the National Statement on Ethical Conduct in Human Research (2007; Updated December 2018). The Chief Investigators were responsible for reporting any SAEs to the relevant Ethics Committee as soon as possible, and in accordance with the guidelines of the Ethics Committee. When an AE/SAE occurred, start and stop dates, action taken, outcome, and relationship to study treatment (causality) were documented.

Supplementary information- Consent process

Where possible written informed consent was obtained directly from the participant directly, by a trained trial therapist (but not one who would be providing usual care) and witnessed by someone that did not have any involvement in the research project. In some instances, however, the trial therapist was responsible for both providing usual care services, and the trial intervention as they were the only one available to conduct the consent process. There were also circumstances that meant that it was not possible to have a completely impartial witness present at the time of informed consent (such as if consent occurred at the participants home). In such circumstances a family member or other person who was known to the participant was required to act as witness. There were also circumstances where the person with aphasia was unable to provide written consent (i.e., because they are unable to read or write due to post-stroke complications) and in such cases then their verbal consent was obtained and the consent process was witnessed by an impartial witness (i.e., no relation or connection to the researcher). If verbal rather than written consent was obtained from the participant, a note detailing the date and time that verbal consent occurred was written on the consent form. Once the person with aphasia had given informed consent, participants with aphasia were asked if they would like a family member to be involved in the study and to nominate this family member. If the family member agreed, the speech pathologist verbally explained the study as well as provided family members with a participant information sheet and obtain their written informed consent. All potential participants were informed that participation in the research project was voluntary and that if they choose to participate or not participate in the project, it would not negatively impact upon the medical care that they received. Potential participants were informed that they are free to withdraw from the study at any point without any adverse consequences. The informed consent process used aphasia friendly information and allowed supported communication tools to aid the participants capacity to consent. Speech pathologists trained in the consent process made a clinical judgement about the person’s capacity to consent. If it could not be determined that consent was freely given with supported communication, participants were not included.

**Table 1: CONSORT 2010 checklist of information to include when reporting a cluster randomised trial**

| Section/Topic | Item No | Standard Checklist item | Extension for cluster designs | Page No * |
| --- | --- | --- | --- | --- |
| Title and abstract | | | |  |
|  | 1a | Identification as a randomised trial in the title | Identification as a cluster randomised trial in the title | Preventing depression in aphasia: a cluster randomised control trial of the Aphasia Action Success Knowledge (ASK) program |
|  | 1b | Structured summary of trial design, methods, results, and conclusions (for specific guidance see CONSORT for abstracts)^[[1]](#endnote-1),^^[[2]](#endnote-2)^ | See table 2 | Background: Stroke patients with aphasia and their caregivers have higher incidence of depression than those without aphasia.  Aims: To determine whether a tailored intervention program (Action Success Knowledge; ASK) led to better mood and quality of life (QoL) outcomes than an attention control with a 12 month end point at cluster and individual participant level.  Methods: A multi-site, pragmatic, two-level single blind cluster randomized controlled trial compared ASK to an attention control (secondary stroke prevention program). Ten metropolitan and 10 non-metropolitan health regions were randomized. People with aphasia and caregivers were recruited within 6 months post-stroke who scored ≤12 on the Stroke Aphasic Depression Questionnaire Hospital Version-10 at screening. Each arm received manualised intervention over 6-8 weeks followed by monthly telephone calls. Blinded assessments of QoL and depression were taken at 12 months post-onset.  Results: Twenty clusters (health regions) were randomised. Trained speech pathologists screened 1744 people with aphasia and 373 participants consented to intervention (n=231 people with aphasia and 142 family members). Multilevel mixed effects modelling under the intention to treat protocol showed a significant difference on the Stroke and Aphasia Questionnaire-21 (SADQ-21 N=122, 17 clusters) in favour of the attention control. Individual data analysis using a minimal detectable change score for the SADQ-21 showed the difference was not meaningful.  Conclusion: ASK showed no benefit over attention control in improving mood and preventing depression in people with aphasia or their family members.  Funding acknowledgement: National Health and Medical Research grant APP1060673  Trial registration: Australian Clinical Trials [ACTRN12614000979651](https://www.australianclinicaltrials.gov.au/anzctr/trial/ACTRN12614000979651) |
| Introduction | | | |  |
| Background and objectives | 2a | Scientific background and explanation of rationale | Rationale for using a cluster design | to reduce the risk of treatment contamination and to enable provision of study interventions alongside usual care for the study duration (i.e., across multiple health facilities within the first year post-stroke). |
|  | 2b | Specific objectives or hypotheses | Whether objectives pertain to the the cluster level, the individual participant level or both | Hypothesis 1: Patients with aphasia who receive the ASK program will have significantly better outcomes on the Stroke and Aphasia Depression Questionnaire (SADQ-21) and the Assessment for Living with Aphasia (ALA) at 12 months post stroke than those who receive an attention control program.  Hypothesis 2: Caregivers of patients with aphasia who receive the ASK program will have significantly better outcomes on the Bakas Caregiver Outcomes Scale (BCOS) and the General Health Questionnaire-28 (GHQ-28) at 12 months post stroke than those who receive an attention control program. |
| Methods | | | |  |
| Trial design | 3a | Description of trial design (such as parallel, factorial) including allocation ratio | Definition of cluster and description of how the design features apply to the clusters | Clusters for the study were Australian Health Service Districts that offer speech pathology rehabilitation services. Health Service Districts were chosen (as opposed to individual hospital sites) so that the study interventions could be provided alongside the usual continuum of care for the study duration (that is, across multiple health facilities within the first-year post stroke) |
|  | 3b | Important changes to methods after trial commencement (such as eligibility criteria), with reasons |  | The eligibility criteria throughout the trial were changed to enable attempts to recruit the full sample size target. The following changes were made:  • If the screening date was on or after the 14.11.2016 the exclusion criteria that required the person to have a first incidence of post stroke aphasia no longer applied.  • If the screening date was on or after the 13.02.2017 the exclusion criteria about previous depression history no longer applied.  • If the screening date was on or after the 13.02.2017 there was an increase to the cut-off score for entry into the study on our depression screening tool SADQH-10 from 9 to 12. In other words, people who scored 11 or less on the SADQ-H were eligible to enter the study. |
| Participants | 4a | Eligibility criteria for participants | Eligibility criteria for clusters | The inclusion criterion for clusters was that they must have provided aphasia rehabilitation services, with the capacity to provide services over the period of intervention. Clusters were excluded if participants were enrolled in other clinical trials at the time of randomization, which limited the recruitment capacity and/or conflicted with the intervention requirements of the current trial. |
|  | 4b | Settings and locations where the data were collected |  | At a location convenient to the participant, health site or home |
| Interventions | 5 | The interventions for each group with sufficient details to allow replication, including how and when they were actually administered | Whether interventions pertain to the cluster level, the individual participant level or both | 7- Clusters (speech pathologists trained in either the attention control or experimental intervention that was administer to individuals with aphasia and their family members)  TIDER checklist included |
| Outcomes | 6a | Completely defined pre-specified primary and secondary outcome measures, including how and when they were assessed | Whether outcome measures pertain to the cluster level, the individual participant level or both | 6 individual level  Two primary outcomes for people with aphasia mood as measured by the Stroke Aphasic Depression Questionnaire- 21 item (SADQ-21) and quality of life as measured by the Assessment for Living with Aphasia (ALA) [  The secondary outcome for people with aphasia included a 10-item measure of self-reported stroke risk-related behaviours. Both ideal (for example, taking medication as prescribed) and nonideal behaviours (for example, smoking cigarettes) will be measured with higher scores out of 10 indicating performance of more ideal behaviours. Secondary outcomes for family members of people with aphasia was the impact of caregiving measured by the Bakas Caregiving Outcomes Scale Revised (BCOS) and mental health as measured by the General Health Questionnaire-28 item (GHQ-28).. |
|  | 6b | Any changes to trial outcomes after the trial commenced, with reasons |  | No changes |
| Sample size | 7a | How sample size was determined | Method of calculation, number of clusters(s) (and whether equal or unequal cluster sizes are assumed), cluster size, a coefficient of intracluster correlation (ICC or *k*), and an indication of its uncertainty | 5 Sample size calculations were calculated for both primary outcome measures (ALA and SADQ-21). The ALA required a larger sample size compared to the SADQ-21, and therefore, the larger sample size required by the ALA was determined necessary to adequately power the study. To achieve a power of 80 % with a 5 % level of significance in comparing the two arms of the study (Aphasia ASK versus attention control - SSPIP), we need 186 patients (93 per arm) with an effect size of 0.367, computed using ALA data. The extent to which power is diminished by clustering was considered in relation to the design effect (DE) = 1 + (m -1)r10, where m = the average size of a cluster and r is the intra-class correlation coefficient. Typically, intraclass correlation coefficients are small (<0.02); thus a conservatively estimated intra-class correlation of 0.02 was used. A cluster size of 20 was chosen based on the feasibility of running the intervention, as well as the availability of patients with aphasia within clusters. Thus DE = 1+ (20-1)*0.02 = 1.38, and the total sample size required was calculated as 186 * 1.38 ≈ 258. To account for an attrition rate of 25 % to the 12-month follow-up period, 344 patients was needed (172 per arm). |
|  | 7b | When applicable, explanation of any interim analyses and stopping guidelines |  | **N/a** |
| Randomisation: | | | |  |
| Sequence generation | 8a | Method used to generate the random allocation sequence |  | 5 Clusters were randomly allocated in progressive blocks (2 capital, 2 noncapital) by the blinded statistician (AK) to either the experimental arm (the ASK program) or the similarly formatted and delivered attention control arm (SSPIP), using a computer-generated random number scheme. Sequential numbers were assigned to each cluster to ensure allocation concealment. |
|  | 8b | Type of randomisation; details of any restriction (such as blocking and block size) | Details of stratification or matching if used | 5 Stratification based on location: Urban clusters or capital city (n = 10) and Nonurban clusters or non-capital city (n = 10) |
| Allocation concealment mechanism | 9 | Mechanism used to implement the random allocation sequence (such as sequentially numbered containers), describing any steps taken to conceal the sequence until interventions were assigned | Specification that allocation was based on clusters rather than individuals and whether allocation concealment (if any) was at the cluster level, the individual participant level or both | 5 concealment was individual level Allocation concealment from cluster site staff was not possible as each of the intervention arms had different content. |
| Implementation | 10 | Who generated the random allocation sequence, who enrolled participants, and who assigned participants to interventions | Replace by 10a, 10b and 10c | 5 blinded statistician (AK); clusters enrolled participants |
|  | 10a |  | Who generated the random allocation sequence, who enrolled clusters, and who assigned clusters to interventions | 5 |
|  | 10b |  | Mechanism by which individual participants were included in clusters for the purposes of the trial (such as complete enumeration, random sampling) | 5 |
|  | 10c |  | From whom consent was sought (representatives of the cluster, or individual cluster members, or both), and whether consent was sought before or after randomisation | 6 consent from participants sought after cluster randomisation |
|  |  |  |  |  |
| Blinding | 11a | If done, who was blinded after assignment to interventions (for example, participants, care providers, those assessing outcomes) and how |  | 5,6  Participants with aphasia, their family and outcome assessors were blinded to intervention allocation. |
|  | 11b | If relevant, description of the similarity of interventions |  | 7 The attention control arm of the study (SSPIP) was intended to be provided in a similar dosage and format to the experimental intervention. The SSPIP condition attempted to control for the attention and time provided by speech pathologists to participants |
| Statistical methods | 12a | Statistical methods used to compare groups for primary and secondary outcomes | How clustering was taken into account | 7 A multilevel mixed-effects model, that took into account participants being nested within clusters, examined whether changes in the continuous outcomes of interest varied over time and across the two groups, after adjustment for potential confounders. For binary outcomes, mixed effects logistic regression was used to examine the effects of the intervention. Covariates included age, gender, educational level, change in living situation during the intervention, discontinuation of usual care speech pathology services during the intervention period, walking status at the start of the intervention, and baseline severity of aphasia. |
|  | 12b | Methods for additional analyses, such as subgroup analyses and adjusted analyses |  | 8 Per Protocol analysis was undertaken that included only those participants who had completed more than 3 or more face to face sessions, had both pre-and post-measures collected and where intervention was delivered by a therapist who passed the treatment fidelity check. Adherence to the phone calls was not considered in the Per Protocol analysis as there was little compliance to this prescribed treatment component. To determine if significant differences were meaningful, individual participant data analysis was undertaken21 using the most relevant psychometric data from Sutcliffe & Lincoln, 199812. For the SADQ-21 a minimal detectable change (MDC) value of 9.45 was used. |
| Results | | | |  |
| Participant flow (a diagram is strongly recommended) | 13a | For each group, the numbers of participants who were randomly assigned, received intended treatment, and were analysed for the primary outcome | For each group, the numbers of clusters that were randomly assigned, received intended treatment, and were analysed for the primary outcome | Supplementary file Figure 1 |
|  | 13b | For each group, losses and exclusions after randomisation, together with reasons | For each group, losses and exclusions for both clusters and individual cluster members | Supplementary file Figure 1 |
| Recruitment | 14a | Dates defining the periods of recruitment and follow-up |  | **8** |
|  | 14b | Why the trial ended or was stopped |  | 8- funding expended |
| Baseline data | 15 | A table showing baseline demographic and clinical characteristics for each group | Baseline characteristics for the individual and cluster levels as applicable for each group | Table 1. p9 |
| Numbers analysed | 16 | For each group, number of participants (denominator) included in each analysis and whether the analysis was by original assigned groups | For each group, number of clusters included in each analysis | Supplementary file Figure 1 |
| Outcomes and estimation | 17a | For each primary and secondary outcome, results for each group, and the estimated effect size and its precision (such as 95% confidence interval) | Results at the individual or cluster level as applicable and a coefficient of intracluster correlation (ICC or k) for each primary outcome | Table 3, p13 |
|  | 17b | For binary outcomes, presentation of both absolute and relative effect sizes is recommended |  |  |
| Ancillary analyses | 18 | Results of any other analyses performed, including subgroup analyses and adjusted analyses, distinguishing pre-specified from exploratory |  | Table 3, p13. |
| Harms | 19 | All important harms or unintended effects in each group (for specific guidance see CONSORT for harms^[[3]](#endnote-3)^) |  | 14, Supplementary Table C |
| Discussion | | | |  |
| Limitations | 20 | Trial limitations, addressing sources of potential bias, imprecision, and, if relevant, multiplicity of analyses |  | **14** major biases include an insufficient sample size bias (sample size of 181 instead of 344 required via power analysis), collider bias (the attention control intervention had a probable effect on outcome, the ASK intervention was more complex for the participants to understand), and insensitive measure bias (both primary outcomes measures were self report). Other potential biases may include a recall bias (outcomes were reported 12 months post-onset which may have been several months post intervention), and ascertainment bias (the correct identification of individuals to be recruited to the study such that participants were included who did not have depression or were low mood). |
| Generalisability | 21 | Generalisability (external validity, applicability) of the trial findings | Generalisability to clusters and/or individual participants (as relevant) | Hypothesis not supported |
| Interpretation | 22 | Interpretation consistent with results, balancing benefits and harms, and considering other relevant evidence |  | **14, 15** This cluster RCT showed no measurable benefit of ASK intervention, which was designed to improve mood and QoL outcomes for the person with aphasia and their family member. Both hypotheses that the ASK program would benefit participants more than the attention control were not supported |
| Other information | | |  |  |
| Registration | 23 | Registration number and name of trial registry |  | **3** Trial registration: Australian Clinical Trials ACTRN12614000979651 |
| Protocol | 24 | Where the full trial protocol can be accessed, if available |  | **4** https://trialsjournal.biomedcentral.com/articles/10.1186/s13063-016-1257-9#Tab1 |
| Funding | 25 | Sources of funding and other support (such as supply of drugs), role of funders |  | 3 National Health and Medical Research grant APP1060673 |

** Note: page numbers optional depending on journal requirements*

1. Hopewell S, Clarke M, Moher D, Wager E, Middleton P, Altman DG, et al. CONSORT for reporting randomised trials in journal and conference abstracts. *Lancet* 2008, 371:281-283 [↑](#endnote-ref-1)
2. Hopewell S, Clarke M, Moher D, Wager E, Middleton P, Altman DG at al (2008) CONSORT for reporting randomized controlled trials in journal and conference abstracts: explanation and elaboration. *PLoS Med* 5(1): e20 [↑](#endnote-ref-2)
3. Ioannidis JP, Evans SJ, Gotzsche PC, O'Neill RT, Altman DG, Schulz K, Moher D. Better reporting of harms in randomized trials: an extension of the CONSORT statement. *Ann Intern Med* 2004; 141(10):781-788.

   **
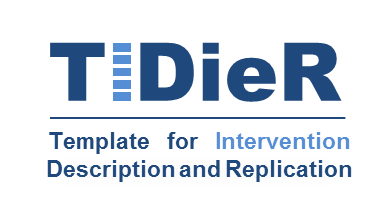
The TIDieR (Template for Intervention Description and Replication) Checklist*:**

   Information to include when describing an intervention and the location of the information

   | **Item number** | **Item** | **Where located **** | |
   | --- | --- | --- | --- |
   |  |  | Primary paper  (page or appendix  number) | Other ^†^ (details) |
   |  | **BRIEF NAME** |  |  |
   | **1.** | Provide the name or a phrase that describes the intervention. | Title + Abstract | ______________ |
   |  | **WHY** |  |  |
   | **2.** | Describe any rationale, theory, or goal of the elements essential to the intervention. | Pg. 7 | _____________ |
   |  | **WHAT** |  |  |
   | **3.** | Materials: Describe any physical or informational materials used in the intervention, including those provided to participants or used in intervention delivery or in training of intervention providers. Provide information on where the materials can be accessed (e.g. online appendix, URL). | URL mentioned on pg 7 | https://www.latrobe.edu.au/research/centres/health/aphasia/resources |
   | **4.** | Procedures: Describe each of the procedures, activities, and/or processes used in the intervention, including any enabling or support activities. | URL mentioned on pg 7 | In _____________ |
   |  | **WHO PROVIDED** |  |  |
   | **5.** | For each category of intervention provider (e.g. psychologist, nursing assistant), describe their expertise, background and any specific training given. | pg 6 | Training resources available at URL |
   |  | **HOW** |  |  |
   | **6.** | Describe the modes of delivery (e.g. face-to-face or by some other mechanism, such as internet or telephone) of the intervention and whether it was provided individually or in a group. | Pg 6 | _____________ |
   |  | **WHERE** |  |  |
   | **7.** | Describe the type(s) of location(s) where the intervention occurred, including any necessary infrastructure or relevant features. | Pg 6 | _____________ |
   |  | **WHEN and HOW MUCH** |  |  |
   | **8.** | Describe the number of times the intervention was delivered and over what period of time including the number of sessions, their schedule, and their duration, intensity or dose. | Supplementary Tables A & B | _____________ |
   |  | **TAILORING** |  |  |
   | **9.** | If the intervention was planned to be personalised, titrated or adapted, then describe what, why, when, and how. | n/a | _____________ |
   |  | **MODIFICATIONS** |  |  |
   | **10.^ǂ^** | If the intervention was modified during the course of the study, describe the changes (what, why, when, and how). | n/a | _____________ |
   |  | **HOW WELL** |  |  |
   | **11.** | Planned: If intervention adherence or fidelity was assessed, describe how and by whom, and if any strategies were used to maintain or improve fidelity, describe them. | Pg 5 | _____________ |
   | **12.^ǂ^** | Actual: If intervention adherence or fidelity was assessed, describe the extent to which the intervention was delivered as planned. | Supp Table B | _____________ |

   ** **Authors** - use N/A if an item is not applicable for the intervention being described. **Reviewers** – use ‘?’ if information about the element is not reported/not sufficiently reported.

   † If the information is not provided in the primary paper, give details of where this information is available. This may include locations such as a published protocol or other published papers (provide citation details) or a website (provide the URL).

   ǂ If completing the TIDieR checklist for a protocol, these items are not relevant to the protocol and cannot be described until the study is complete.

   * We strongly recommend using this checklist in conjunction with the TIDieR guide (see *BMJ* 2014;348:g1687) which contains an explanation and elaboration for each item.

   * The focus of TIDieR is on reporting details of the intervention elements (and where relevant, comparison elements) of a study. Other elements and methodological features of studies are covered by other reporting statements and checklists and have not been duplicated as part of the TIDieR checklist. When a **randomised trial** is being reported, the TIDieR checklist should be used in conjunction with the CONSORT statement (see [www.consort-statement.org](http://www.consort-statement.org)) as an extension of **Item 5 of the CONSORT 2010 Statement.** When a **clinical trial** **protocol** is being reported, the TIDieR checklist should be used in conjunction with the SPIRIT statement as an extension of **Item 11 of the SPIRIT 2013 Statement** (see [www.spirit-statement.org](http://www.spirit-statement.org)). For alternate study designs, TIDieR can be used in conjunction with the appropriate checklist for that study design (see [www.equator-network.org](http://www.equator-network.org)). [↑](#endnote-ref-3)
